# The Relationship Between Social–Emotional Skills and the Perception of School Violence Among Chilean Children and Adolescents

**DOI:** 10.3390/bs16071084

**Published:** 2026-07-01

**Authors:** Flavio Muñoz-Troncoso, Ricardo García-Hormazábal, Enrique Riquelme-Mella, Rhys Allardice, Isabel Cuadrado-Gordillo, Gerardo Muñoz-Troncoso

**Affiliations:** 1 Faculty of Education, Universidad Católica de Temuco, Temuco 4810296, Chile; flavio.munoz@uct.cl; 2 Faculty of Social Sciences and Arts, Universidad Mayor, Temuco 4801043, Chile; 3 Faculty of Education, Pontificia Universidad Católica de Chile, Santiago 8331150, Chile; rallardice0@estudiante.uc.cl; 4 Faculty of Education and Psychology, Universidad de Extremadura, 06071 Badajoz, Spain; cuadrado@unex.es; 5 Faculty of Philosophy and Humanities, Universidad Austral de Chile, Valdivia 5110566, Chile; gerardo.munoz01@uach.cl

**Keywords:** social–emotional skills, school adjustment, childhood and adolescence, school violence, perception, profiles

## Abstract

This study examines the relationship between social–emotional skills (SES) and perceptions of school violence among primary school students (3rd through 8th grade), considering both associations and heterogeneity in social–emotional profiles. A quantitative, non-experimental, cross-sectional design was used, with a sample of 311 students aged 8 to 15 (M = 10.65, SD = 1.69). SES were assessed across four dimensions (stress management, adaptation, sense of safety, and expectations), while perceptions of school violence included verbal, physical, relational, digital, and teacher-perpetrated acts. The results show that all dimensions of SES have significant inverse associations with perceptions of violence, with moderate magnitudes, suggesting their role as cognitive–emotional resources. Likewise, three distinct SES profiles (high, medium, and low; n = 151, 134, and 26, respectively) were identified, with the profile exhibiting the highest levels generally reporting lower perceptions of school violence. However, differences between profiles do not follow a simple linear pattern, suggesting more complex, non-incremental dynamics. Nevertheless, no differences in the perception of violence were found based on gender, despite evident differences in SES. The results indicate that SES operate as situated functional systems that modulate the interpretation of violence. It is concluded that understanding school violence requires integrating variable- and profile-centred approaches and considering the interaction between individual resources and school contexts.

## 1. Introduction

The return to in-person schooling following the pandemic marked a turning point in school violence levels in Chile, which have shown a sustained increase through the end of 2025 (SUPEREDUC, 2025). However, the mechanisms through which students experience and interpret this violence remain poorly understood. In this regard, social–emotional skills (SES) have emerged as a central analytical focus in contemporary educational research, as they provide insight into the processes through which children and adolescents interpret, regulate, and respond to the demands of the school environment. Previous literature has defined SES as integrated cognitive–affective and relational systems that organise social experiences in specific contexts (Arango et al., 2024; Denham et al., 2015; Eisenberg et al., 2010). From this perspective, SES play a key role in social interpretation processes, directly shaping how individuals interpret ambiguous situations, regulate their emotional responses, and select strategies for action (Compas et al., 2017; Crick & Dodge, 1994; Herrera-Pulgarín, 2025; Lemerise & Arsenio, 2000).

This interpretive dimension takes on particular significance in the study of school violence. Traditionally approached from a perspective centred on direct physical aggression, school violence has gradually been reconceptualised as a relational, contextual, and multidimensional phenomenon that includes verbal, relational, symbolic, and digital forms (Espelage & Swearer, 2010; Hong & Espelage, 2012; Menesini & Salmivalli, 2017). A significant step forward has been the recognition of its perceptual nature: the experience of violence does not depend solely on its objective occurrence, but also on the processes through which students interpret social interactions (Thornberg, 2015; Thornberg et al., 2017). Thus, two students exposed to similar situations may report significantly different levels of perceived violence, making subjective variability a central component of the phenomenon. This perceptual variability is systematically associated with differences in socioemotional resources: higher levels of emotional regulation, adaptive coping, and perceived social support are associated with lower levels of perceived victimisation, even under comparable conditions of exposure (Wang et al., 2026). However, the evidence suggests that these relationships do not follow strictly linear patterns, which poses challenges for traditional explanatory models (Cantor et al., 2019).

In this vein, a persistent limitation in the literature is the prevalence of variable-centred approaches, which assume homogeneous relationships between SES and psychosocial outcomes. Nevertheless, recent research has challenged this premise, showing that socioemotional development is organised into heterogeneous configurations or profiles that combine different skills in a non-uniform manner, leading to distinct trajectories of adjustment, vulnerability, and resilience (Cantor et al., 2019; Masten et al., 2021; Mulvey et al., 2022; Zych et al., 2021). This shift toward person-centred approaches provides a more accurate understanding of the complexity of social–emotional development and its impact on the school experience, particularly in phenomena that depend on interpretive processes, such as the perception of violence. Within this person-centred perspective, variables traditionally considered explanatory, such as gender, have shown limited scope in accounting for these dynamics, given the high intragroup variability and the significant overlap of profiles (Hyde, 2014; Zell et al., 2015).

Despite these advances, a gap remains between models that analyse the direct effects of SES on perceptions of school violence and those that address the heterogeneity of socioemotional profiles. This disconnection limits understanding of the mechanisms by which SES shape distinct school experiences, thereby reducing the explanatory power of current models.

In this context, the present study aims to examine the relationship between social–emotional skills and perceptions of school violence among primary school students (3rd through 8th grade), while simultaneously considering (a) the direct effects of social–emotional competencies and (b) the heterogeneity in social–emotional development profiles. This approach seeks to contribute to a more integrated understanding of the phenomenon by articulating complementary analytical levels and moving toward models that recognise the complexity of the processes shaping the experience of violence in school settings.

### 1.1. Social–Emotional Skills and Development in School Settings

SES are a key multidimensional construct for understanding how children and adolescents interpret, regulate, and respond to the demands of the school environment. In contrast to reductionist approaches focused on isolated traits, contemporary research conceptualises them as integrated systems of cognitive–affective and relational processes that enable individuals to recognise and regulate their own emotions, understand the experiences of others, and act adaptively in complex social contexts (CASEL, 2015; Jones et al., 2011; OECD, 2024). From this perspective, SES do not function as static attributes, but rather as situated functional resources whose activation depends on contextual characteristics and the specific demands of social interaction (Avivar-Cáceres et al., 2022; Denham et al., 2015; Eisenberg et al., 2010).

This situated nature is essential to understanding their role in the school experience, given that SES mediate social interpretation processes such as the attribution of intentionality, the assessment of threat, and the regulation of emotional responses to ambiguous or potentially conflictual situations (Crick & Dodge, 1994; Fuentes-Vilugrón et al., 2025; Lemerise & Arsenio, 2000). Therefore, SES constitute social processing systems that organise students’ subjective experiences within contexts of everyday interactions (Denham et al., 2015; Eisenberg et al., 2010; Lemerise & Arsenio, 2000; Muñoz-Troncoso & Riquelme-Mella, 2025).

Recent literature has also emphasised the need to distinguish among skills, competencies, and socioemotional dispositions without empirically separating them. While skills refer to specific abilities that can be learned, such as emotional regulation or conflict resolution, competencies involve their functional integration into real-world contexts, and dispositions refer to relatively stable tendencies that influence their activation and use (Duckworth & Yeager, 2015; Kankaraš & Suárez-Álvarez, 2019). These dimensions form an interdependent system in which skills emerge based on individual dispositions and are consolidated into competencies through social experience, thereby reinforcing a non-reductionist view of social–emotional development.

This system takes on particular importance during middle childhood and early adolescence, a period characterised by significant changes in emotional regulation, social cognition, and interpersonal sensitivity (Blair & Raver, 2015; Dahl et al., 2018; Verdugo, 2026). At this stage, the strengthening of executive functions leads to greater self-regulation, while the growing centrality of peer groups increases the complexity of relational dynamics and the demands for psychosocial adjustment (Somerville, 2013). However, recent evidence challenges homogeneous evolutionary models, showing that the development of SES is characterised by multiple, asynchronous trajectories, with high levels of intra- and inter-individual heterogeneity (Howard & Ferrari, 2022; Masten & Cicchetti, 2016).

This heterogeneity stems from the interaction of individual, relational, and contextual factors, including school experiences, the quality of relationships with peers and adults, and varying levels of exposure to psychosocial stressors (Cantor et al., 2019; Jones et al., 2019). Thus, students with similar levels of exposure to adverse situations may differ significantly in how they interpret those experiences, a distinction that is particularly relevant for understanding complex relational phenomena such as school violence.

In this context, while the literature has documented gender differences in certain dimensions of social–emotional learning, such as higher levels of empathy in girls, recent studies suggest that these differences have limited explanatory power, given the high degree of intragroup variability and the overlap of social–emotional profiles (Hyde, 2014; Zell et al., 2015).

From a functional perspective, SES can be understood as psychosocial systems that bridge the relationship between the school environment and students’ subjective experiences (Cantor et al., 2019; Compas et al., 2001; Osher et al., 2020). Factors such as emotional regulation, active coping, perceived social support, and positive expectations interact in an integrated manner, shaping distinct patterns of adjustment and adaptation (Compas et al., 2017; Domitrovich et al., 2017; Osher et al., 2020).

### 1.2. School Violence as a Relational Phenomenon

School violence has been traditionally approached from a behavioural perspective focused on direct physical aggression; however, in recent decades, there has been a conceptual shift toward ecological approaches that view it as a relational, contextual, and multidimensional phenomenon (Espelage & Swearer, 2010; Hong & Espelage, 2012). Olweus’s pioneering work (Olweus, 1993) established fundamental criteria such as repetition and power asymmetry, but contemporary research has significantly expanded this framework by incorporating symbolic, relational, and digital dimensions of violence.

Today, school violence encompasses not only physical acts but also verbal, relational, and structural forms, such as social exclusion, humiliation, symbolic violence, and cyberbullying (Menesini & Salmivalli, 2017; Muñoz-Troncoso et al., 2022; Thornberg, 2015; Utley et al., 2022). These forms of violence operate within complex systems of interaction, where social norms, group hierarchies, and institutional dynamics shape students’ day-to-day experiences.

In this context, it is essential to recognise that school violence cannot be understood solely in terms of its objective occurrence, but necessarily involves its perceptual dimension (Hong & Espelage, 2012; Meldrum et al., 2022; Thornberg, 2015; Thornberg et al., 2017). The experience of violence depends on processes of social interpretation, including the attribution of intent, the assessment of threat, and the interpretation of implicit norms of interaction (Thornberg, 2015).

Thus, different forms of violence—verbal, physical, relational, digital, and perpetrated by adults—have distinct yet converging effects on emotional well-being, perceptions of safety, and a sense of belonging at school (Gaffney et al., 2021; Zych et al., 2021). Furthermore, these forms often coexist and interact, creating cumulative experiences of victimisation that intensify psychosocial impact. From this perspective, school violence is understood as a situated relational phenomenon, the understanding of which requires integrating individual, social, and institutional dimensions. This approach opens the door to analysing not only the risk factors associated with violence but also the protective mechanisms that modulate its perception and impact, with SES playing a central role.

In light of the above, it can be argued that SES provide a robust explanatory framework for understanding individual differences in the perception of school violence, insofar as these systems shape the interpretation, evaluation, and response to social interactions. Recent evidence indicates that students with higher levels of emotional regulation, adaptive coping, and perceived social support tend to interpret ambiguous situations as less threatening, reporting lower levels of perceived victimisation even in objectively adverse contexts (Espelage & Hong, 2019; Muñoz-Troncoso & Riquelme-Mella, 2025; Wang et al., 2026). These findings suggest that SES not only influence behavioural responses to violence, but also the cognitive–affective processes that shape perceptions of it.

SES are organised into distinct profiles, in which specific combinations of skills are associated with different levels of vulnerability or resilience to school violence (Mulvey et al., 2022; Zych et al., 2021). In this regard, the perception of violence emerges from the dynamic interaction between contextual conditions and individual socioemotional configurations, which helps explain why students with similar levels of exposure report divergent subjective experiences.

This perspective is particularly relevant for primary school students (3rd through 8th grade), a stage marked by heightened social awareness and increasingly complex peer relationships. In this context, students with uneven socioemotional development may amplify perceptions of threat, exclusion, or injustice, whereas those with a more balanced development tend to employ interpretive and relational strategies that mitigate the psychosocial impact of violence (Gómez-Villalpando & González-García, 2026).

Consequently, analysing the relationship between SES and perceptions of school violence requires considering not only the direct effects of social–emotional competencies, but also the diversity of profiles and developmental trajectories that shape varied school experiences. Within this framework, the present study aims to examine the relationship between social–emotional skills and perceptions of school violence among primary school students, considering both the direct effects of social–emotional competencies and the heterogeneity in student profiles. To this end, the following hypotheses are proposed:

**H1.**  *Dimensions of social–emotional skills will show statistically significant inverse associations with the perception of school violence.*

**H2.**  *Gender differences in social–emotional skills will be reflected in the corresponding differences in perceptions of school violence.*

**H3.**  *Distinct student profiles will be identified based on their social–emotional skills.*

**H4.**  *At least one profile with higher levels of SES will be identified that reports a lower perception of school violence compared to profiles with lower SES.*

It is worth noting that H2 is proposed as an exploratory hypothesis. While the literature has documented gender differences in socioemotional variables and perceptions of violence, partly linked to historically rooted structural inequalities, recent evidence suggests that contextual, social, and cultural factors substantially moderate these differences, limiting their direct explanatory power (Hyde, 2014; Zell et al., 2015). H2 therefore seeks to examine whether these differences are expressed in this specific context, without presupposing their confirmation.

## 2. Materials and Methods

The study employs a quantitative, non-experimental, cross-sectional, and comparative methodology (León & Montero, 2015; McMillan & Schumacher, 2005).

### 2.1. Participants

A total of 311 students from the school system, aged 8 to 15 years (M = 10.65; SD = 1.69), participated. Of the total participants, 55.6% were male, and 44.4% were female. All collected cases were retained for analysis; no participants were excluded. At the time of participation, the students were in grades 3 through 8 (Table 1).

### 2.2. Instruments

A Social–Emotional Skills Questionnaire (SES) was developed for this study, consisting of 16 items that measure four dimensions: [1] stress management (4 items, e.g., “I have been able to calm myself down when something makes me sad or angry”); [2] adaptation (4 items, e.g., “When I’ve had a problem, I try to solve it calmly”); [3] sense of security (4 items, e.g., “I’ve felt that my family protects me”); and [4] expectations (4 items, e.g., “A lot of good things happen to me during the day”). The dimensions are conceptually aligned with different constructs: the stress management dimension relates to models of emotional regulation (Gross & John, 2003); the adaptation dimension relates to active coping strategies, specifically problem-solving and seeking social support (Ayers et al., 1996); the security dimension relates to scales of perceived family social support (Zimet et al., 1988); and the expectations dimension relates to constructs of dispositional optimism (Ortiz et al., 2016; Scheier & Carver, 1985). The items are rated on a 6-point Likert scale from 1 (Strongly Disagree) to 6 (Strongly Agree), with higher scores indicating higher levels of SES.

The Abbreviated CENVI Questionnaire (Muñoz-Troncoso et al., 2023), specifically its second-order factor ‘Types of School Violence’ (TSV), validated as a unifactorial model by Muñoz-Troncoso and Riquelme-Mella (2025), was also used. This is a scale that measures students’ perceptions of types of school violence, composed of five first-order dimensions: [1] verbal violence (3 items, e.g., “Some students give their classmates irritating nicknames”); [2] physical violence (3 items, e.g., “Some students fight near the school”); [3] exclusion (3 items, e.g., “Some classmates embarrass or ridicule others to damage their image”); [4] cyberbullying (3 items, e.g., “Some students create social media accounts under fake names to spread comments that embarrass others”); and [5] teacher-perpetrated violence (3 items, e.g., “Some teachers grab students firmly by the arms to get their attention”). The scale demonstrated a good fit in the confirmatory factor analysis (CFA) (Muñoz-Troncoso et al., 2023; Muñoz-Troncoso & Riquelme-Mella, 2025), with internal reliability indices ω > 0.80 across all dimensions, and scalar invariance for gender, ethnicity, administrative dependency, and grade level (Muñoz-Troncoso et al., 2023). It is a 6-point Likert scale (1 = Never–6 = Always) where higher scores indicate a greater perception of TSV.

### 2.3. Procedure

School administrators were contacted to obtain active informed consent from parents online through a platform that guaranteed their identification. Parents were initially responsible for informing their children about the study. Students provided informed assent prior to participation. To ensure comprehension across all age levels, adults read and explained the assent form to the students, which had been previously reviewed and adapted for easy understanding relative to their age. Participation was voluntary, and students were informed of their right to withdraw at any time without consequence. The procedure was carried out on the school premises, and each administration was supervised by two adults: a professional from the school’s coexistence department and the course’s homeroom teacher. Additionally, members of the research team were intermittently present at the application site. The presence of these professionals maintained classroom order and ensured that the teachers’ role did not influence the students’ individual responses.

The survey is hosted on a LimeSurvey-based platform (LimeSurvey GmbH, 2024) available at www.censa.cl. It begins with a description of the study, an informed assent form, and a privacy notice that includes details about the research, the survey, and the estimated time required to complete it. The platform was configured to require a response to each item before proceeding, preventing missing data. Students’ voluntary participation was emphasised, with the option to withdraw at any time by simply closing the browser window; anonymity and data protection were guaranteed. Given the strictly anonymous and digital nature of the platform, the instrument did not identify specific individual cases. However, due to the presence of the homeroom teacher and the school coexistence professional during the application, any acts of violence or student deregulation would immediately trigger the school’s internal protocols, which align with Chilean educational coexistence regulations.

The study was conducted in accordance with the international ethical guidelines set forth in the Declaration of Helsinki (World Medical Association, 2013) and the Declaration of Singapore (Resnik & Shamoo, 2011), as well as with the Chilean regulations established in Law 20.120 (MINSAL, 2006).

### 2.4. Plan of Analysis

The SES scale underwent content validation through expert judgment, with Aiken’s V coefficient (Aiken, 1980) calculated to assess the appropriateness, relevance, and clarity of the items. A value of V ≥ 0.70 was considered adequate (Penfield & Giacobbi, 2004). For both scales, the normality of all variables (items and mean scores per dimension) was examined in order to select the estimators and hypothesis tests for subsequent analyses. The SES scale was also subjected to CFA.

Although recent studies have demonstrated the validity, reliability, and scalar invariance of the CENVI questionnaire across various categories (Muñoz-Troncoso et al., 2022, 2023), it was deemed appropriate to also review the goodness-of-fit of the indices, as was done for the SES scale, using a CFA. This was done as a preliminary step to the analysis of the proposed structural relationship, which was conducted using structural equation modelling (SEM). Thus, for both CFA and SEM, the unweighted least squares mean- and variance-adjusted estimator (ULSMV) was used (Brown, 2015; Muthén & Muthén, 2017). To assess the model’s goodness-of-fit (in CFA and SEM), the following were considered:

(1) The chi-square statistic and its ratio to the degrees of freedom (χ^2^/df); (2) the Root Mean Square Error of Approximation (RMSEA); (3) the Comparative Fit Index (CFI); and (4) the Tucker–Lewis Index (TLI). Theoretically, a model is considered to have an adequate fit when the χ^2^/df ratio is equal to or less than 3; the RMSEA is less than 0.08—ideally less than 0.05; and the CFI and TLI indices are equal to or greater than 0.90—and preferably 0.95 (Byrne, 2016; Hu & Bentler, 1999; Kline, 2023). The reliability of each dimension was assessed using McDonald’s omega coefficient (McDonald, 1999), with values ≥0.60 considered acceptable for exploratory research (Hair et al., 2019).

For the SES scale, evidence of construct validity was examined. To this end, convergent validity was assessed through the average variance extracted (AVE), with values greater than 0.50 considered adequate. Discriminant validity was evaluated using the Fornell–Larcker criterion (Fornell & Larcker, 1981), whereby the square root of the AVE for each dimension must exceed its correlation with the remaining dimensions. Since discriminant validity was not met, and given that the construct has theoretical support for its four-dimensional structure, a second-order factor model was estimated (Lévy & Varela, 2006; Varela et al., 2006). This decision was not driven solely by statistical criteria, but primarily by the construct’s conceptual nature. The dimensions evaluated do not function in isolation; rather, they operate as theoretically interconnected components. Therefore, estimating a second-order model provides a more realistic reflection of this hierarchical structure, as the methodological literature supports that first-order dimensions can share a common theoretical foundation (Hair et al., 2019; Kline, 2023).

The structural relationship between SES and the perception of TSV was assessed using the gamma coefficient (γ). For interpretation, thresholds of 0.10 (low), 0.30 (moderate), and 0.50 (high) were used (Cohen, 1988). Statistical significance was set at *p* < 0.05 (Hair et al., 2019). Furthermore, to examine the associations between each SES dimension and the second-order TSV factor, Spearman correlations were calculated. For interpretation, thresholds of 0.10 (low), 0.30 (moderate), and 0.50 (high) were used. Statistical significance was set at *p* < 0.05.

In addition, differences between men and women in SES dimensions and perceptions of TSV were explored using Mann–Whitney U tests. This analysis enabled examination of whether gender is a significant source of heterogeneity in the patterns observed between the two constructs. Statistical significance was set at *p* < 0.05.

In addition, a two-stage cluster analysis was conducted on the SES scales to identify latent student profiles based on their socioemotional competence. This approach allows for an examination of the sample’s heterogeneity beyond linear relationships between variables (Kaufman & Rousseeuw, 1990). Schwarz’s Bayesian Information Criterion (BIC) was used to determine the optimal number of clusters, and the log-likelihood measure was employed as the distance metric (Chiu et al., 2001). Subsequently, differences in TSV perceptions across the identified profiles were assessed. The Kruskal–Wallis test was applied to evaluate global differences across each dimension of violence. For dimensions yielding significant results, pairwise Mann–Whitney U tests were conducted, applying the Bonferroni correction within each dimension family (*m* = 3) to control for Type I error, with statistical significance set at *p* < 0.05.

The analyses were performed using MPlus v.8.8 (Muthén & Muthén, 2024), SPSS v.25 (IBM Corp., 2020) and Excel v16.74 (Microsoft, 2023).

## 3. Results

Content validation of the SES questionnaire was conducted with seven experts. Aiken’s V coefficients ranged from 0.78 to 1 for relevance, from 0.81 to 1 for significance, and from 0.75 to 0.98 for clarity. All items met the criterion of V ≥ 0.70. The Kolmogorov–Smirnov test showed that the data do not follow a normal distribution (*p* < 0.001). Regarding the CFAs, a good fit of the proposed model to the data was found for both the SES scale (χ^2^ = 254.557; df = 98; RMSEA = 0.070; CFI = 0.951; TLI = 0.940) and the TSV scale (χ^2^ = 160.914; df = 85; RMSEA = 0.054; CFI = 0.975; TLI = 0.969).

The composite reliabilities of the SES dimensions, along with the convergent and discriminant validity evidence for the scale, are presented in Table 2.

**Figure 1:**
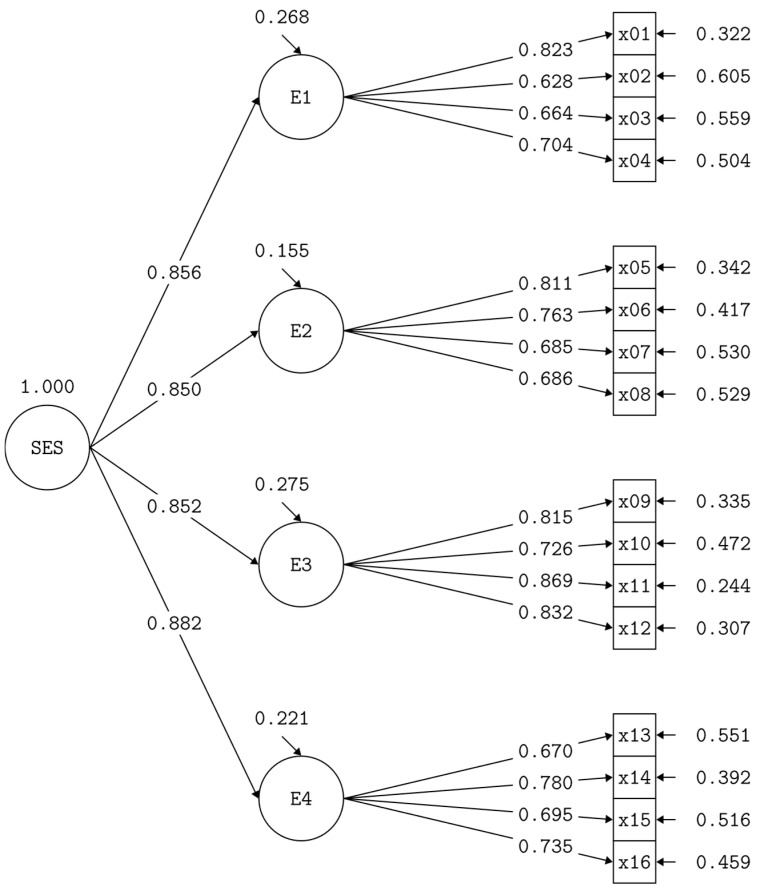
Second-Order Confirmatory Factor Analysis Model for the SES Scale. Note: First-order latent variables for SES: E1 = Stress, E2 = Adaptation, E3 = Safety, E4 = Expectation. Source: Prepared by the authors.

Although scale reliability and convergent validity were achieved, the lack of discriminant validity, together with the theoretical support for the four dimensions of the SES scale, led us to estimate a second-order model. This structure showed a good fit (χ^2^ = 268.777; df = 100; RMSEA = 0.072; CFI = 0.947; TLI = 0.937). Further details on the model’s characteristics are shown in Figure 1.

For TSV, these results were: verbal violence (ω = 0.820), physical violence (ω = 0.822), exclusion (ω = 0.864), cyberbullying (ω = 0.883), and teacher violence (ω = 0.860).

**Figure 2:**
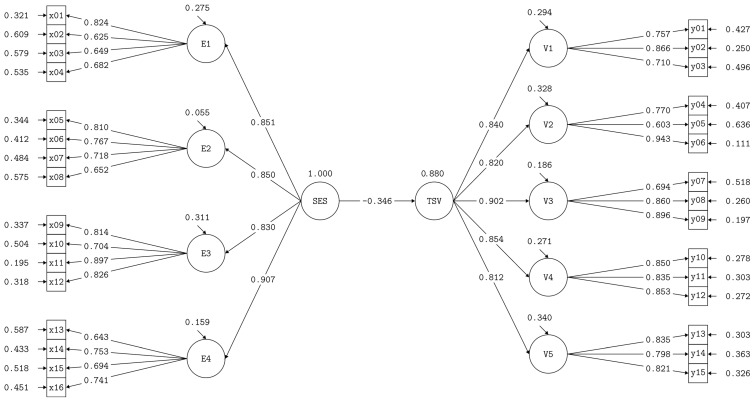
Structural Equation Model for the Effect of SES on TSV. Note: First-order latent variables for TSV: V1 = Verbal, V2 = Physical, V3 = Exclusion, V4 = Digital, V5 = Teacher-perpetrated. Source: Prepared by the authors.

The SEM analysis revealed that the proposed model fit the data well (χ^2^ = 587.726, df = 424, RMSEA = 0.035, CFI = 0.960, TLI = 0.956). Furthermore, a statistically significant moderate effect was observed between the SES second-order factor and the TSV second-order factor (γ = −0.346; *p* < 0.001; 95% CI [−0.459, −0.234]). A more detailed representation of this structural relationship is illustrated in Figure 2.

Correlations among the SES scale dimensions and TSV are presented in Table 3.

The comparison by gender showed that men scored significantly higher on stress management (*p* = 0.011), safety (*p* = 0.044), and expectations (*p* = 0.045). The difference in adaptation did not reach statistical significance, although it was marginally above the threshold (*p* = 0.055). Table 4 presents the detailed statistics.

Regarding perceptions of TSV, no statistically significant differences were found between men and women in any of the five dimensions assessed (*p* > 0.05). However, men reported higher average scores on all TSV measures. Table 5 presents the detailed statistics.

A two-stage cluster analysis using the four dimensions of SES identified three profiles based on Schwarz’s Bayesian information criterion (BIC = 568.027). The quality of the clustering was satisfactory (silhouette coefficient = 0.6). Profile 1 (n = 151; 48.6%) was characterised by high scores on all SES dimensions. Profile 2 (n = 134; 43.1%) had intermediate scores. Profile 3 (n = 26; 8.4%) had the lowest scores on all dimensions. Statistically significant differences were found in the distribution of profiles by gender (χ^2^ = 7.563, df = 2, *p* = 0.023), with a higher proportion of men in Profile 1 (63.6% men vs. 36.4% women), while Profile 2 (48.5% men vs. 51.5% women) and Profile 3 (46.2% men vs. 53.8% women) both showed a higher proportion of women. Table 6 presents the detailed centroids for each profile.

The Kruskal–Wallis test for independent samples revealed statistically significant differences across all TSV dimensions between the profiles (Table 7).

Mann–Whitney U tests were conducted for each TSV dimension across profile pairs. Following Bonferroni correction, statistically significant differences were found between Profile 1 and Profile 2 in four of the five dimensions—verbal violence, exclusion, digital violence, and teacher-perpetrated violence—with Profile 2 reporting higher perceptions of violence; physical violence did not reach significance after correction. Comparisons between Profile 1 and Profile 3 revealed a significant difference in only one dimension: exclusion, with Profile 3 reporting higher perceptions. No statistically significant differences were found between Profile 2 and Profile 3 in any TSV dimension. Table 8 presents the detailed statistics for these comparisons.

## 4. Discussion

The aim of this study was to examine the relationship between social–emotional skills (SES) and perceptions of school violence among primary school students (3rd through 8th grade). Overall, the results suggest that SES are associated with how students interpret relational dynamics in the school environment.

First, the findings of H1 are consistent with the literature that conceptualises SES as systems associated with the cognitive–affective evaluation of social experiences (Ordoñez Rodríguez, 2022; Osher et al., 2020; Panayiotou et al., 2019). All dimensions of SES showed statistically significant inverse associations with perceptions of school violence. Although effect sizes at the dimensional level were small, the overall structural association between the second-order SES factor and TSV showed a moderate magnitude. This pattern suggests that students with greater resources for emotional regulation, coping, perceived support, and positive expectations tend to interpret school interactions as less threatening, even in potentially adverse contexts.

This pattern is consistent with the multidimensional nature of the perception of school violence, which does not depend exclusively on individual variables, but rather on the interaction between personal, relational, and institutional factors (Malti et al., 2018; Taylor et al., 2017). Accordingly, SES do not eliminate exposure to violence but rather are associated with the interpretive processes through which it is perceived and evaluated.

One relevant finding is that the expectations dimension showed the strongest association. This finding suggests that dispositional optimism and the anticipation of positive outcomes constitute particularly relevant resources for the reinterpretation of ambiguous events, reducing the attribution of harmful intent and the perception of threat (Compas et al., 2017). This result highlights the importance of considering anticipatory cognitive dimensions within intervention models for school coexistence.

Regarding H2, this hypothesis was not confirmed: the results indicate that, although gender differences exist in some dimensions of SES, these do not translate into differences in the perception of school violence. This finding suggests that gender, as a categorical variable, has limited explanatory power in this type of phenomenon, which is consistent with evidence highlighting high intragroup variability and the overlap of socioemotional profiles (Hyde, 2014; Löffler & Greitemeyer, 2023). Consequently, gender appears to play an indirect role, contributing to the configuration of socioemotional profiles rather than directly explaining differences in the perception of violence.

In this regard, findings regarding H3 and H4 provide particularly relevant evidence. The identification of three distinct SES profiles–high, medium, and low–and their association with different levels of perceived violence reinforces the need to move from variable-centred models toward person-centred approaches. The finding that the profile with the highest levels of SES generally reports lower perceptions of violence is consistent with the literature describing these competencies as resources associated with lower perceptions of psychosocial vulnerability (Domitrovich et al., 2017; Zych et al., 2021).

However, the results also suggest that this relationship is not entirely linear. Differences between profiles are consistent when contrasting high and medium levels of SES, but they do not reach statistical significance when comparing medium and low levels. This lack of linearity is primarily explained by the smaller sample size of the lowest profile, which limits the statistical power to detect subtle differences between these groups. Therefore, these findings must be interpreted strictly in a descriptive manner for this sample, highlighting the need for future studies with larger subgroups to accurately evaluate the behaviour of the lower levels of the construct.

It is important to consider certain limitations. As mentioned above, the small sample size of the lowest SES profile limits the statistical power to detect subtle differences between groups, suggesting that the absence of statistically significant differences involving Profile 3 should be interpreted with caution, as some comparisons reached significance prior to correction. Moreover, the study’s cross-sectional design precludes causal inference; therefore, future research should incorporate longitudinal designs to examine the directionality of these relationships.

Regarding the psychometric properties of the SES scale, it should be acknowledged that the instrument is in an initial stage of validation. Although the second-order model is theoretically grounded in the interconnection of its dimensions and provided an adequate statistical fit for this sample, the questionnaire was developed specifically for this study. Therefore, since it has not been tested in an independent or larger sample to consolidate this hierarchical structure in other populations, the generalisability of these results should be limited.

From a theoretical perspective, integrating the SES dimensions under a unified second-order structure represents an ongoing conceptual challenge within the current literature in the field of psychology. Although this model offers an adequate empirical and conceptual solution to reflect the interconnection of the variables, the discussion persists as to whether these components should be interpreted as a global, cohesive socioemotional construct or rather as a set of related yet functionally independent psychosocial resources. Acknowledging this theoretical ambiguity enriches future discussions and highlights the need for further research to more precisely delineate the nature of these resources.

Finally, the results suggest that SES operate as functional systems of interpretation rather than as static attributes, and that their association with the perception of school violence is organised in a heterogeneous and context-dependent manner (Carmona-Rojas et al., 2023). This reinforces the need to design interventions that strengthen individual competencies, taking into account the relational and structural dynamics of the school environment.

## 5. Conclusions

The results suggest that social–emotional skills (SES) are inversely associated with perceptions of school violence, with effect sizes ranging from low at the dimensional level to moderate at the level of the overall SES construct.

Furthermore, the findings highlight the need to complement variable-centred approaches with person-centred approaches. The identification of distinct profiles and their nonlinear patterns with perceptions of violence reinforces the importance of considering the heterogeneity of social–emotional development.

Regarding gender, the results suggest an indirect role, calling into question its use as a linear explanatory variable and highlighting its role in the configuration of social–emotional profiles rather than in the direct perception of violence.

However, these results must be interpreted with certain limitations in mind. Although the SES questionnaire demonstrated adequate reliability, convergent validity, and theoretical coherence within the proposed second-order structure, it was specifically developed for this study and therefore should be considered a preliminary measurement approach. The cross-sectional design prevents the establishment of causal relationships between variables, while the small size of some subgroups, particularly those with lower socioemotional development, limits the statistical power to detect finer differences. Furthermore, the use of self-reported measures may introduce biases associated with social desirability or subjective interpretation of the items.

Based on the above, future research should continue to examine psychometric properties of SES in independent samples, particularly regarding discriminant validity and the stability of its higher-order structure across different educational contexts. Likewise, the incorporation of longitudinal designs to examine the directionality of the relationship between SES and perceptions of violence, as well as expanding analysis to different sociocultural and educational contexts, may provide further understanding. Similarly, it is important to further investigate profile-centred approaches and explore the role of contextual variables, such as school climate, teaching practices, and community dynamics, in shaping these relationships. Finally, it is suggested that future research move toward integrated models that link socioemotional interventions with institutional strategies to simultaneously address individual interpretive processes and the structural conditions that underpin school violence.

## Figures and Tables

**Table 1:** Distribution of participants by grade level.

Grade	Frequency	Percentage	Cumulative Percentage
3rd grade	56	18.0	18.0
4th grade	48	15.4	33.4
5th grade	53	17.0	50.5
6th grade	56	18.0	68.5
7th grade	60	19.3	87.8
8th grade	38	12.2	100.0
Total	311	100.0	

Source: Prepared by the authors.

**Table 2:** Reliability, Convergent Validity, and Discriminant Validity of the SES Scale.

	AVE	ω	Stress	Adaptation	Safety	Expectation
Stress	0.502	0.823	0.709			
Adaptation	0.545	0.852	0.895	0.738		
Safety	0.660	0.885	0.646	0.896	0.812	
Expectation	0.508	0.805	0.751	0.842	0.774	0.713

Note: On the diagonal, the square root of the AVE. Below the diagonal, correlations between factors. Source: Prepared by the authors.

**Table 3:** Spearman Correlations Between SES Dimensions and TSV.

Variable SES	*r*_s_	*p*	L	U
Stress	−0.242	<0.001	−0.347	−0.131
Adaptation	−0.172	0.002	−0.281	−0.059
Safety	−0.206	<0.001	−0.313	−0.094
Expectation	−0.271	<0.001	−0.374	−0.161

Note: Correlation is significant at the 0.01 level. Source: Prepared by the authors.

**Table 4:** Differences between men and women on the SES scale.

	Gender				
Variable Socioemotional	Male N = 173 Mean Rank	Female N = 138 Mean Rank	U	Z	*p*
Stress management	167.60	141.46	9930.50	−2.555	0.011
Adaptation	164.68	145.12	10,436.00	−1.915	0.055
Safety	165.01	145.12	10,377.50	−2.016	0.044
Expectation	165.10	144.59	10,362.00	−2.007	0.045

Source: Prepared by the authors.

**Table 5:** Differences between men and women in their perception of TSV.

	Gender				
Variable Type of Violence	Male N = 173 Mean Rank	Female N = 138 Mean Rank	U	Z	*p*
Verbal	159.77	151.27	11,284.50	−0.831	0.406
Physical	162.38	148.01	10,834.00	−1.421	0.155
Exclusion	159.79	151.25	11,281.00	−0.840	0.401
Digital	161.43	149.19	10,997.00	−1.236	0.217
Teacher-perpetrated	162.73	147.57	10,773.00	−1.643	0.100

Source: Prepared by the authors.

**Table 6:** Centroids of the SES profiles.

Dimension	Profile 1 (n = 151)	Profile 2 (n = 134)	Profile 3 (n = 26)	Combined (N = 311)
	M (SD)	M (SD)	M (SD)	M (SD)
Stress management	4.94 (0.64)	3.76 (0.82)	2.76 (0.99)	4.25 (1.04)
Adaptation	5.48 (0.40)	4.47 (0.59)	2.65 (1.11)	4.81 (0.99)
Safety	5.75 (0.33)	5.04 (0.71)	2.56 (1.11)	5.18 (1.05)
Expectations	5.19 (0.55)	4.21 (0.78)	2.86 (1.41)	4.57 (1.03)

Source: Prepared by the authors.

**Table 7:** Kruskal–Wallis Test for TSV Dimensions Across SES Profiles.

Type of Violence	Test Statistic	*p*
Verbal	12.474	0.002 *
Physical	6.184	0.045 **
Exclusion	16.555	<0.001 *
Digital	15.416	<0.001 *
Teacher-perpetrated	23.718	<0.001 *

Note: * Statistical significance at the 0.01 level; ** Statistical significance at the 0.05 level. Source: Prepared by the authors.

**Table 8:** Mann–Whitney U Test for TSV Dimensions Across SES Profiles.

Type of Violence	Profile	Mean Rank	Profile	Mean Rank	U	Z	*p_adj_*
Verbal	Profile 1	127.76	Profile 2	160.17	7816.0	−3.32	0.003 **
	Profile 1	85.79	Profile 3	107.63	1478.5	−2.02	0.132
	Profile 2	80.09	Profile 3	82.62	1687.0	−0.26	1
Physical	Profile 1	133.40	Profile 2	153.81	8668.0	−2.12	0.102
	Profile 1	86.23	Profile 3	105.10	1544.5	−1.77	0.228
	Profile 2	79.26	Profile 3	86.90	1575.5	−0.78	1
Exclusion	Profile 1	126.36	Profile 2	161.75	7604.0	−3.65	0.003 **
	Profile 1	84.87	Profile 3	112.96	1340.0	−2.63	0.027 *
	Profile 2	78.94	Profile 3	88.56	1532.5	−0.97	0.990
Digital	Profile 1	126.19	Profile 2	161.94	7578.5	−3.79	0.003 **
	Profile 1	85.94	Profile 3	106.75	1501.5	−2.03	0.129
	Profile 2	80.99	Profile 3	77.98	1676.5	−0.31	1
Teacher-perpetrated	Profile 1	122.91	Profile 2	165.64	7083.5	−4.87	0.003 **
	Profile 1	86.43	Profile 3	103.92	1575.0	−1.95	0.153
	Profile 2	81.53	Profile 3	75.17	1603.5	−0.67	1

Note 1: *p*_adj_ = Adjusted *p*-value using Bonferroni correction. Note 2: * Significance at the 0.05 level after Bonferroni correction and ** Significance at the 0.01 level after Bonferroni correction. Source: Prepared by the authors.

## Data Availability

The data presented in this study are available on request from the corresponding author.
